# The role of *Drosophila *Merlin in spermatogenesis

**DOI:** 10.1186/1471-2121-9-1

**Published:** 2008-01-10

**Authors:** Natalia V Dorogova, Elena M Akhmametyeva, Sergei A Kopyl, Natalia V Gubanova, Olga S Yudina, Leonid V Omelyanchuk, Long-Sheng Chang

**Affiliations:** 1 Institute of Cytology and Genetics, Russian Academy of Sciences, Novosibirsk, Russia; 2 Center for Childhood Cancer, Research Institute at Nationwide Children's Hospital and Department of Pediatrics, The Ohio State University College of Medicine, Columbus, Ohio, USA

## Abstract

**Background:**

*Drosophila *Merlin, the homolog of the human *Neurofibromatosis 2 *(*NF2*) gene, is important for the regulation of cell proliferation and receptor endocytosis. Male flies carrying a *Mer*^3 ^allele, a missense mutation (Met^177^→Ile) in the *Merlin *gene, are viable but sterile; however, the cause of sterility is unknown.

**Results:**

Testis examination reveals that hemizygous *Mer*^3 ^mutant males have small seminal vesicles that contain only a few immotile sperm. By cytological and electron microscopy analyses of the *Mer*^3^, *Mer*^4 ^(Gln^170^→stop), and control testes at various stages of spermatogenesis, we show that *Merlin *mutations affect meiotic cytokinesis of spermatocytes, cyst polarization and nuclear shaping during spermatid elongation, and spermatid individualization. We also demonstrate that the lethality and sterility phenotype of the *Mer*^4 ^mutant is rescued by the introduction of a wild-type *Merlin *gene. Immunostaining demonstrates that the Merlin protein is redistributed to the area associated with the microtubules of the central spindle in telophase and its staining is less in the region of the contractile ring during meiotic cytokinesis. At the onion stage, Merlin is concentrated in the Nebenkern of spermatids, and this mitochondrial localization is maintained throughout sperm formation. Also, Merlin exhibits punctate staining in the acrosomal region of mature sperm.

**Conclusion:**

*Merlin *mutations affect spermatogenesis at multiple stages. The Merlin protein is dynamically redistributed during meiosis of spermatocytes and is concentrated in the Nebenkern of spermatids. Our results demonstrated for the first time the mitochondrial localization of Merlin and suggest that Merlin may play a role in mitochondria formation and function during spermatogenesis.

## Background

Spermatogenesis is a model that facilitates studies of the effect of gene mutations on mitosis, meiosis and the remodeling of many cell structures. During spermatogenesis, primordial germ cells undergo an oriented mitotic division to replace themselves and to produce spermatogonia (reviewed in [1,2]). Each spermatogonium undergoes four rounds of mitotic division, generating 16 spermatogonia that subsequently differentiate into spermatocytes within a cyst. Since the cytokinesis of mitotic divisions is incomplete, the spermatogonia are connected by ring channels. Then, all 16 spermatocytes go through two rounds of meiotic divisions, resulting in a cyst of 64 haploid, round-shaped spermatids. The meiotic cytokinesis is also incomplete so that the spermatids remain interconnected.

During the coalescence stage in early spermatids, the mitochondria aggregate to the side of the nucleus, where the centriole resides [2]. By the onion stage of spermatid differentiation, a dramatic transformation of the mitochondrial mass occurs. The individual mitochondria fuse into two giant mitochondria, which are arranged in a densely-packed sphere consisting of many layers of wrapped mitochondrial membranes [1]. This onion-like structure is termed the Nebenkern.

At the elongation stage, the flagellar axoneme elongates, resulting in a dramatic change in the shape of the spermatid [1]. The two interlocked subunits of the Nebenkern unfold and extend along with the growing axoneme. Despite the structural changes of the two mitochondrial derivatives, both mitochondrial subunits remain aligned and associated with axoneme. As spermatids begin to elongate, their heads, containing nuclei, remain aligned toward the testis wall and their tails are turned aside toward the testicular apex. Simultaneously, the cyst slides down along the testis wall, changing its shape from a disc-like structure to a bundle of elongating spermatids with the nuclear regions of spermatids polarized toward the base of the testis [3]. Following the flagellar elongation, the spermatid nucleus transforms its shape from a spherical structure to a long, thin needle. Subsequently, the process of individualization is initiated by the formation of the individualization complex (IC), containing the actin cones at the head region of the spermatid bundle [4]. Individualization occurs in a cystic bulge, progressing along the entire length of the spermatid bundle. During individualization, membrane remodeling takes place, the channels connecting spermatids are destroyed, and syncytial organization of a cyst is lost [5]. Following coiling of the sperm bundle, mature sperm are released into the testis lumen and then pass into the seminal vesicle.

Although the spermatogenesis process has been well defined, only a limited number of genes whose mutations affect this process has been described, and the role of their protein products are mostly unknown. *Mer*^3^, a mutation (Met^177^→Ile) in the gene encoding *Drosophila *Merlin, whose ortholog in human is named the *Neurofibromatosis 2 *(*NF2*) gene [6,7], leads to male sterility [8]. Male flies with a *Mer*^3 ^allele are viable but sterile; however, the cause of sterility is unknown.

Merlin shares a high degree of homology to the ezrin, radixin, and moesin (ERM) proteins, which belong to the protein 4.1 superfamily, linking the actin cytoskeleton to the plasma membrane [9]. Interaction of the ERM proteins with the actin cytoskeleton is thought to be important for the determination of the cell-shape, cell adhesion, cell motility, cytokinesis, and intracellular signaling [10,11]. In addition to its interaction with the actin cytoskeleton, Merlin can bind to microtubules and regulate the microtubule cytoskeleton [12,13]. Studies in mammalian cells show that Merlin mediates contact inhibition of proliferation [14]. Merlin inactivation leads to tumor formation in several cell types in mammals [15].

Merlin is evolutionally conserved [16]. The Merlin homolog in *Drosophila melanogaster *shows extensive sequence homology to the human protein [17]. This similarity between the fly and human Merlin proteins extends over the entire amino acid sequences with the greatest similarity in the amino terminus of the FERM domain (F for protein 4.1) [9,16-18]. The homology between the fly and human Merlin proteins also exists in the carboxyl terminus, a region in which Merlin diverges from the ERM-family members [10,16].

The *Drosophila Merlin *gene (*D-Mer*) is located at the 18D-E region of the X chromosome. *D-Mer *has been shown to regulate cell proliferation and survival through the Hippo signaling pathway [8,19-23]. In addition, Merlin promotes endocytosis of several membrane signaling receptors [24,25]. Furthermore, *D-Mer *is non-autonomously required to maintain polarity of posterior follicle cells in the oocyte and to limit their proliferation [26].

To better understand the cause of sterility in Merlin mutant flies, we carried out an extensive analysis of cellular events in spermatogenesis. We showed that the Merlin protein was concentrated in the mitochondrial derivatives, and that *Merlin *mutations affected meiosis, cyst polarization, nuclear shaping, and axoneme-Nebenkern association.

## Results

### The sterility phenotype in Merlin mutants is rescued by the introduction of a wild-type Merlin gene

**Figure 1:**
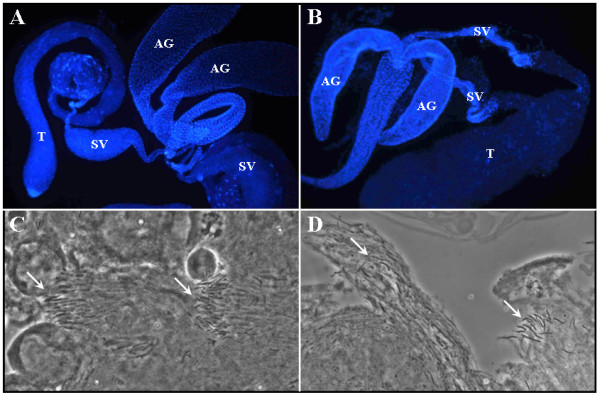
Morphological examination of the testis tissues from young wild-type (panels A and C) and *Mer*^3 ^(panels B and D) males. The control *FM6 *and *Mer*^3 ^mutant males were obtained according to Materials and Methods. The DAPI-stained testis from the control male contained a bulging seminal vesicle (SV) (A). In contrast, a shriveled seminal vesicle was seen in the Mer^3 ^testis (B). T, testis; AG, accessory gland. Acetic acid/orcein staining revealed that the testis tissue from the control male contained well-organized sperm bundles (arrows) (C), while the Mer^3 ^testis had fewer sperm in a more disorganized bundle (arrows) (D).

The *Mer*^3 ^allele carries a missense mutation (Met^177^→Ile), and male flies hemizygous for *Mer*^3 ^are viable but sterile [8]. Upon examination of the testis, we noted that seminal vesicles from the *Mer*^3 ^males were smaller than those from the control *FM6 *flies (compare Figure 1B with Figure 1A). In addition, very few sperm were found in the *Mer*^3 ^seminal vesicles, and they were immotile, in contrast to those seen in the control *FM6 *siblings obtained from the same cross. Acetic acid/orcein staining showed that the *Mer*^3 ^testis had fewer sperm heads in each bundle than the control testis (compare Figure 1D with Figure 1C). Also, the sperm in the bundle were arranged in a more disorganized fashion.

Previously, LaJeunesse et al. [8] demonstrated that ectopic expression of a *Mer*^+ ^or *Mer*^3 ^transgene using a ubiquitously-expressed Gal4 driver (*T80-Gal4*) in a *Mer*^4 ^(Gln^170^→stop) background rescued *Mer*^4 ^lethality. In addition, insertion of a cosmid construct (*P{cosMer*^+^*}*), carrying the entire *D-Mer *gene, was capable of rescuing various *Mer *mutations. We conducted a similar experiment to test whether a *Mer*^+ ^transgene could rescue the sterility of the *Mer*^4 ^allele. Table 1 shows that males carrying both *Mer*^4 ^and *P{cosMer*^+^*} *were viable and fertile. To ensure germline expression of the *Mer*^+ ^or *Mer*^3^transgene, we cloned the *Mer*^+ ^and *Mer*^3 ^sequences into the pUASP vector, and used them to transform embryos. We showed that both pUASP-*Mer*^+ ^and pUASP-*Mer*^3 ^could rescue the lethality of *Mer*^4 ^mutation when ectopically activated by the *Act5C-Gal4 *driver (Table 1). However, only *Mer*^+ ^over-expression restored the fertility of flies with the *Mer*^4 ^mutation.

**Table 1:** The ability of various transgenes to restore the viability and fertility of the *Mer*^4 ^allele.

**Genetic cross**	**Males carrying *Mer*^4^**	**Viability**	**Fertility**
♀♀ *y w Mer^4^/FM7i, GFP; +/+; +/+ *× ♂♂ *w sn/Y; +/+; P{w^+ ^= cosMer^+^}/+*	*y w Mer^4^/Y; +/+; P{w^+ ^= cosMer^+^}/+*	+	+
	*y w Mer^4^/Y; +/+; +/+*	-	-
♀♀ *y w Mer^4^/M-5, B w^a^; +/+; +/+ *× ♂♂ *y w/Y; Act5C-Gal4/If; P{w^+ ^= UASP-Mer^+^}/TM6, Tb*	*y w Mer^4^/Y; Act5C-Gal4/+; P{w^+ ^= UASP-Mer^+^}/+*	+	+
	*y w Mer^4^/Y; Act5C-Gal4/+; +/TM6, Tb*	-	-
	*y w Mer^4^/Y; If/+; TM6, Tb/+*	-	-
	*y w Mer^4^/Y; If/+; P{w^+ ^= UASP-Mer^+^}/+*	-	-
♀♀ *y w Mer^4^/M-5, B w^a^; +/+; +/+ *× ♂♂ *y w/Y; Act5C-Gal4/If; P{w^+ ^= UASP-Mer^3^}/TM6, Tb*	*y w Mer^4^/Y; Act5C-Gal4/+; P{w^+ ^= UASP-Mer^3^}/+*	+	-
	*y w Mer^4^/Y; Act5C-Gal4/+; +/TM6, Tb*	-	-
	*y w Mer^4^/Y; If/+; TM6, Tb/+*	-	-
	*y w Mer^4^/Y; If/+; P{w^+ ^= UASP-Mer^3^}/+*	-	-

### The Merlin protein is dynamically redistributed during meiosis of spermatocytes and is concentrated in the Nebenkern of spermatids

**Figure 2:**
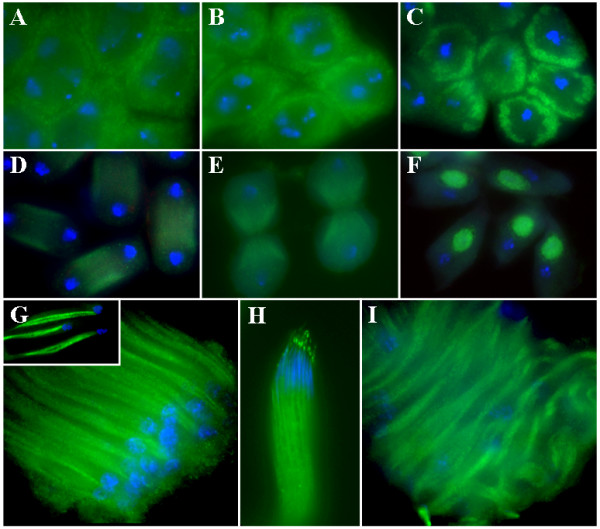
Intracellular distribution of the Merlin protein at various stages of spermatogenesis. In the control cyst, Merlin was detected in the cellular cortex of spermatocytes (A). In prometaphase (B) and metaphase (C) of meiosis, the cortical localization of Merlin became more evident. In telophase (D), Merlin redistributed and accumulated in the area associated with the microtubules of the central spindle. During cytokinesis (E), Merlin staining remained associated with the microtubules but was less intense in the region of the contractile ring. In the onion-stage spermatids (F), Merlin was highly concentrated in the Nebenkern. This localization pattern was maintained through the comet stage of spermatid elongation (G). The insert in panel G shows intense Merlin staining in the two subunits of Nebenkern in spermatids. In the control cyst, containing mature sperm, bright Merlin staining was also seen as a punctate dot in the acrosomal region (H). Merlin staining was still detected in the *Mer*^3 ^cyst at the comet stage; however, sperm nuclei were scattered throughout the cyst, and the arrangement of spermatids was irregular (I).

To understand the cause of sterility in the Merlin mutant flies, we studied the subcellular localization of the Merlin protein in the control *FM6 *and *Mer*^3 ^testes at various stages of spermatogenesis. In the cysts from the control testis, Merlin expression was detected in the cellular cortex of spermatocytes (Figure 2A), as seen in somatic tissues and earlier germ cells [17]. This cortical localization became more pronounced in spermatocytes during prometaphase and metaphase of meiosis (Figures 2B and 2C). Merlin was found to redistribute, and more intense staining was observed in the area associated with the microtubules of the central spindle in telophase (Figure 2D). During cytokinesis, intense Merlin staining remained associated with the microtubules but the intensity was less in the region of the contractile ring (Figure 2E). A similar Merlin distribution pattern was seen during the second meiotic division (data not shown). At the onion stage, Merlin was concentrated in the Nebenkern, a specialized structure formed by the fusion of mitochondria during spermatid differentiation (Figure 2F). This intense Merlin staining in the Nebenkern remained throughout the comet stage of spermatid elongation, during which the Nebenkern split into two parts (Figure 2G). Note that concentrated Merlin immunoreactivity was clearly seen in the two subunits of the Nebenkern in the spermatid (insert in Figure 2G). In the control cyst containing mature sperm, Merlin staining continued to be present in the elongated Nebenkern (Figure 2H). In addition, Merlin was seen as a bright punctate dot in the acrosomal region, a Golgi apparatus-derived structure developed over the anterior part of the sperm's head. We also performed a similar immunostaining on the *Mer*^3 ^testis. Although we could detect Merlin staining in the *Mer*^3 ^cyst at the comet stage, the *Mer*^3 ^spermatid nuclei were scattered throughout the cyst, and the arrangement of spermatids appeared irregular (Figure 2I). The ability of the antibody to detect Merlin staining in the *Mer*^3 ^cyst suggests that the missense mutation in *Mer*^3 ^did not affect antibody recognition. Using the same antibody, LaJeunesse [8] previously detected a similar cortical localization of Merlin in both the wild-type and *Mer*^3 ^imaginal discs. However, the *Mer*^3 ^mutation clearly affects Merlin function as the spermatids in the *Mer*^3 ^cyst were poorly arranged.

### Merlin mutations affect meiotic cytokinesis of spermatocytes, cyst polarization and nuclear shaping during spermatid elongation, and spermatid individualization

**Figure 3:**
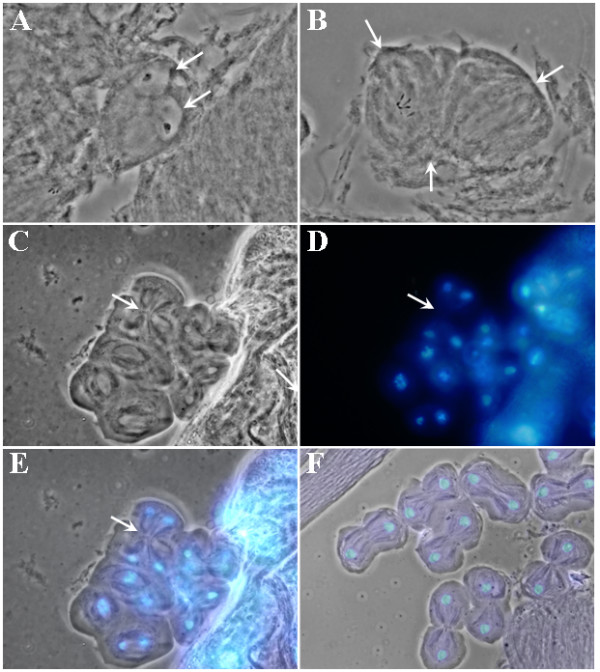
Abnormalities observed during meiosis of Mer^3 ^spermatocytes: a spermatid containing two nuclei of equal size (arrows) and two Nebenkerns (A), tripolar spindle in a spermatocyte going through the second meiotic division (B), and four-polar spindle in a spermatocyte undergoing the second meiotic division (C-E). Panel C shows the phase contrast image, panel D the DAPI-stained nuclei, and panel E the merged image. Arrow points to the central spindle midzone [29]. For comparison, wild-type meiotic cells in telophase were shown (F).

Next, we examined if there were any abnormalities during early steps of spermatogenesis in the *Mer*^3 ^testis. We dissected testes from both the control and *Mer*^3 ^mutant flies. Following staining with acetic acid/orcein, the testes were squashed and examined according to Ashburner [27]. Although we did not find any abnormalities during mitosis of spermatogonia or spermatocyte growth, we observed three types of abnormalities during meiosis of spermatocytes from the *Mer*^3 ^testis, as compared with the control testis. The first type of abnormality is shown in Figure 3A, demonstrating a spermatid containing two nuclei of equal size and two Nebenkerns. This type of abnormality was likely caused by cytokinesis failure during the second meiotic division. The second type of abnormality is tripolar spindle in a spermatocyte going through the second meiotic division (Figure 3B). This result suggests incomplete cytokinesis in the previous meiotic division. The third type of abnormality is four-polar spindle in a spermatocyte undergoing the second meiotic division (Figures 3C–E). Note that a secondary *Mer*^3 ^spermatocyte contained two pairs of telophase nuclei (Figure 3D). Each pair of nuclei was situated with its own spindle, and two spindles shared a common mid zone (Figure 3C and 3E). This is in contrast with wild-type spermatocytes in telophase showing two daughter nuclei separate from each other along the central spindle (Figure 3F). The four-polar spindle represents another case of abnormal cytokinesis in the first meiotic division. It should be mentioned that we detected the first type of abnormality in about 5% of the mutant cysts, while the second and third types of abnormalities appeared less frequent.

**Figure 4:**
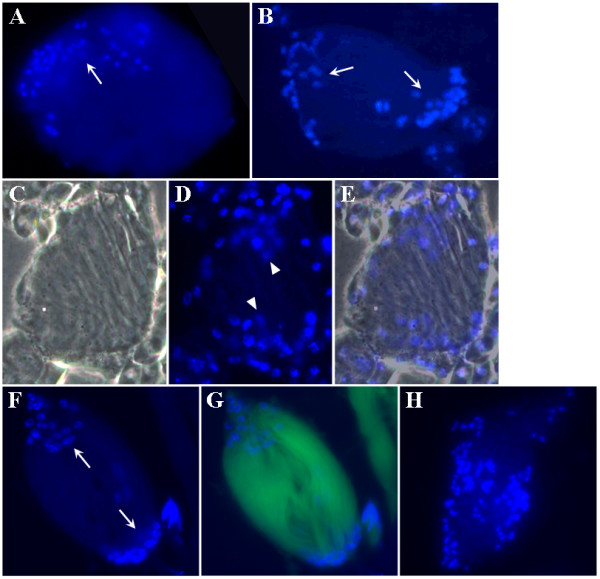
Difference in nuclear grouping during cyst polarization between the control and Merlin mutant spermatids. Dissected testes from the control *FM6 *(A), *Mer*^3 ^(B-E), and *Mer*^4 ^(F-H) males were stained with DAPI and examined as described before. Note that while the spermatid nuclei were grouped in one area (arrow) of the control cyst (A), the spermatid nuclei in the *Mer*^3 ^(B-E) and *Mer*^4 ^(F-G) cysts were seen as two diffuse groups (arrows). In some cases, the spermatid nuclei were scattered in the mutant cyst (H). Panel C shows a phase-contrast image of a *Mer*^3 ^cyst, panel D displays the same cyst stained with DAPI, and panel E represents a merged image. The cyst shown in panels F and G was obtained from a male carrying the *Mer*^4 ^mutation and a GFP marker as described in Materials and Methods.

Following meiosis, spermatid elongation ensues [1]. Prior to spermatid elongation, spermatid nuclei group at a defined area of the cyst wall in a process referred to as cyst polarization [3]. We noted that, at this stage, spermatid nuclei were detected as a group in the control cyst (Figure 4A). Intriguingly, spermatid nuclei in the *Mer*^3 ^cyst were grouped in two locations (Figure 4B–E), and in some other cysts, nuclei appeared more scattered (Figure 4C–E). We also found a similar abnormality in nuclear grouping in the *Mer*^4 ^cyst. Although *Mer*^4 ^is larva-lethal [19], we isolated some rare hemizygous *Mer*^4 ^male pupae from the *y Mer*^4^*/Binsn *stock, and a few of them were able to grow to pharates adults. When examining testis preparations from these *Mer*^4 ^males, we detected spermatid nuclei arranged in two groups (Figures 4F and 4G) or in a scattered manner in all of the mutant cysts (Figure 4H). Also, the cyst polarization defects were more frequently seen in *Mer*^4 ^(about 90%) than *Mer*^3 ^(about 50%) cysts. These results indicate that *Merlin *mutations affect cyst polarization.

**Figure 5:**
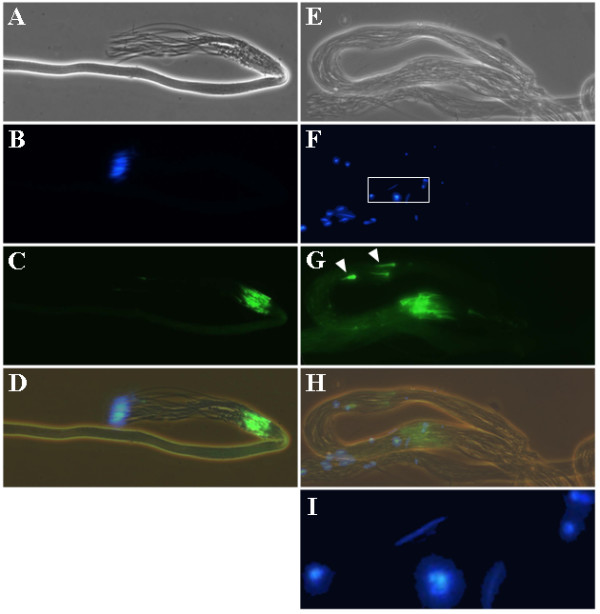
Spermatid individualization in the control (A-D) and *Mer*^3 ^(E-I) cysts. Panels A and E illustrate the phase-contrast view of a control or *Mer*^3 ^cyst, respectively. Panels B and F show the location of the DAPI-stained sperm nuclei. Panels C and G display the sites (arrowheads) and orientation of the actin cones as visualized by FITC-conjugated phalloidin staining. Panels D and H represent merged images. Panel I is an enlarged view of the rectangular area denoted in panel F.

The final stage of spermatogenesis is the process of individualization, followed by sperm coiling [1,2]. The individualization process is initiated at the head of the spermatid cyst, and involves the formation and movement of the actin cones from the head region of the spermatid bundle to the caudal end [4]. Analogous to the previous finding, we detected the actin cones, which moved caudally as a bundle in the control cyst (Figures 5A–D). Note that the sperm heads were grouped in one end of the control cyst, consistent to that seen at the cyst polarization stage (Figures 5B and 5D). Also, the sperm heads became needle-shaped. However, we observed that the sperm nuclei in the *Mer*^3 ^cyst appeared scattered (Figure 5E–H) and had variable morphology; some were round, while others were needle shape (Figure 5I). Unlike the control cyst, the *Mer*^3 ^cyst had the actin cones located at multiple sites (Figure 5G). Dispersed actin cones together with scattered nuclei were also found in the *Mer*^4 ^cyst (data not shown).

### Merlin mutant cysts display abnormalities in Nebenkern-axoneme association

**Figure 6:**
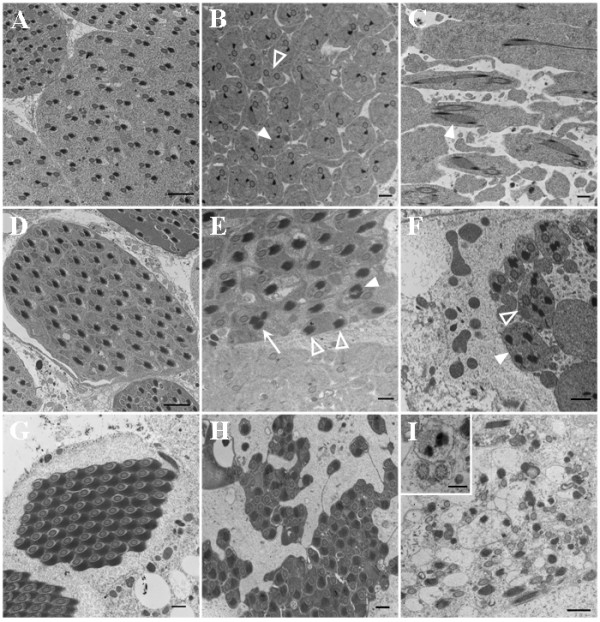
Ultrastructural analysis of the control and Merlin mutant cysts during the elongation and individualization stages. (A-C) Sections of the cysts from the control *FM6 *(A), *Mer*^3 ^(B), or *Mer*^4 ^(C) testis at the elongation stage. (A) A dark paracrystalline body was seen within the major mitochondrial derivative in the control spermatid. Bar = 2 μm. (B) Some of the spermatids in the *Mer*^3 ^cyst contained two paracrystalline bodies (filled arrowhead points to one example). Also, some spermatids had two axonemes (open arrowhead). Bar = 0.5 μm. (C) Two paracrystalline bodies within the major mitochondrial derivative were frequently seen in the elongating spermatids of the *Mer*^4 ^cyst. Bar = 0.5 μm. (D-F) Cysts from the control *FM6 *(D), *Mer*^3 ^(E), or *Mer*^4 ^(F) testis at the individualization stage. (D) The association of axoneme with the mitochondrial derivatives was seen in the spermatids of the control cyst. Bar = 2 μm. (E) Spermatids in the *Mer*^3 ^cyst might contain two abnormally-shaped (filled arrowhead) or three (arrow) paracrystalline bodies together with one axoneme, or have two paracrystalline-filled Nebenkerns but without the axoneme (open arrowhead). Bar = 0.5 μm. (F) Spermatids with multiple Nebenkerns with (open arrowhead) or without (filled arrowhead) axonemes together with cytoplasmic fragmentation were seen in the *Mer*^4 ^cyst. Bar = 0.5 μm. (G-I) Cysts from the control *FM6 *(G), *Mer*^3 ^(H), or *Mer*^4 ^(I) testis at the late stage of individualization. (G) Individualized spermatids in the control cyst displayed a highly-ordered axoneme-Nebenkern pair. Bar = 0.5 μm. (H) The spermatids in the *Mer*^3 ^cyst were poorly arranged and some of them appeared to be fused together. Bar = 0.5 μm. (I) The *Mer*^4 ^cyst showed a complete destruction of spermatid individualization, resulting in empty spermatids with or without axoneme or Nebenkern. Insert illustrates the structure of axoneme appeared to be intact. Bar = 1 μm. Bar in the insert = 0.2 μm.

Since we detected intense Merlin staining in the Nebenkern at the onion stage, we employed electron microscopy to further examine Nebenkern transformation from the structure containing two mitochondrial derivatives at the late stage of spermatid elongation into a configuration filled by electron dense material, called the paracrystalline body, at the end of the individualization stage [4]. Thin sections of testes from the control *FM6*, *Mer*^3^, and *Mer*^4 ^males were analyzed under a transmission electron microscope. As shown in Figure 6A, we observed that at the late elongation stage, each spermatid in the control cyst contained one major and one minor mitochondrial derivative associated with one axoneme. A paracrystalline body could be seen within the major mitochondrial derivative. However, we found that some of the spermatids in the *Mer*^3 ^cyst contained two paracrystalline bodies within the major mitochondrial derivative (Figure 6B). Also, some spermatids had two axonemes. Similarly, two paracrystalline bodies within the major mitochondrial derivative were frequently seen in the elongating spermatids of the *Mer*^4 ^cyst (Figure 6C). It should be mentioned that, unlike the spermatids in the control cyst, which displayed cell-cell contact, the spermatids in the *Mer*^3 ^cyst were loosely arranged (Figures 6B), and those in the *Mer*^4 ^cyst were grossly disorganized (Figure 6C). In addition, some cytoplasmic shedding was present in the *Mer*^3 ^cysts (Figure 6B), and excessive amount of cytoplasmic fragments was seen in the *Mer*^4 ^cyst (Figure 6C).

At the individualization stage, each of the 64 spermatids in the control cyst contained one axoneme associated with the major and minor mitochondrial derivatives (Figure 6D). Furthermore, the paracrystalline body almost filled the entire major mitochondrial derivative. While some spermatids in the *Mer*^3 ^cyst at a similar developmental stage had a paracrystalline body-filled major mitochondrial derivative with axoneme, others containing two paracrystalline body-filled mitochondrial derivatives with abnormal shape or three paracrystalline body-filled mitochondrial derivatives together with one axoneme were also seen (Figure 6E). In addition, spermatids having one or two paracrystalline-filled mitochondrial derivatives but lacking axoneme were observed. Also, the spermatids remained loosely contacted with each other in the *Mer*^3 ^cyst. The paired arrangement of axoneme with the mitochondrial derivatives was often lost in the spermatids of the *Mer*^4 ^cyst at the individualization stage (Figure 6F). Spermatids with multiple paracrystalline body-filled mitochondrial derivatives but without axoneme, or with two axonemes, were found. Also, cytoplasmic fragmentation together with condensed cytoplasmic remnants and gigantic cytoplasmic bodies were also seen in the *Mer*^4 ^cyst.

Consistent with previous observations [1,2,4], we noted that mature spermatids in the control cyst had a substantial reduction in the amount of cytoplasm and a significant reduction in the size of the minor mitochondrial derivative at the end of the individualization stage. The major mitochondrial derivative is filled by a dark-staining paracrystalline material (Figure 6G). Each of the 64 individualized spermatids in the control cyst displayed a highly ordered axoneme-Nebenkern pair. In contrast, a gross disorganization in the arrangement of the individualized spermatids in the *Mer*^3 ^cyst was detected (Figure 6H). Although some spermatids exhibited the axoneme-Nebenkern pair, others appeared to be fused together or connected by a thin cytoplasmic extension. In addition, the boundary between the cysts was not evident, and each cystic area contained less than 64 spermatids. The most dramatic alteration was observed in the *Mer*^4 ^cyst (Figure 6I). Although axoneme and Nebenkern could be found, very little cytoplasmic material was seen, and the cell-cell boundary could not be easily identified. Insert in Figure 6I illustrates that the structure of axoneme appeared to be intact. Despite this dramatic alteration, the 9+2 microtubule-containing structure of axoneme appeared to be preserved in the *Merlin *mutant cysts, indicating that Merlin is not required for axoneme formation and elongation.

Taken together, our results show that Merlin mutations affect cytokinesis, cyst polarization, nuclear shaping, and spermatid individualization. The observation that Merlin is highly concentrated in the Nebenkern suggests that Merlin may pay a role in mitochondria formation and function during various stages of spermatogenesis.

## Discussion

Spermatogenesis is a complicated developmental process, including mitosis, meiosis, cell shape changes, and remodeling of subcellular organelles from the nucleus to mitochondria [1,2]. All of these events appear to involve cytoskeleton reorganization. Merlin has been shown to interact with the actin cytoskeleton and participate in the regulation of cell proliferation, cell adhesion, cell motility, and intracellular signaling [10,11,15]. Merlin can also interact with microtubules and regulate microtubule cytoskeleton [12,13]. In addition, an interaction between Merlin and the myosin heavy chain has been reported [28]. Consistent with findings that Merlin interacts with key components of the cytoskeleton, our results showed that *Merlin *mutations affected meiotic cytokinesis, cyst polarization, nuclear shaping, and spermatid individualization during spermatogenesis. We also showed that the sterility phenotype of hemizygous male Merlin mutants could be rescued by the introduction of a wild-type *Merlin *gene.

The first abnormality that we found in the *Merlin *mutants during spermatogenesis is cytokinesis failure during meiosis of spermatocytes. Cytokinesis is the process of dividing the cytoplasm and separating two daughter cells. It involves the formation of a contractile ring and the central spindle, two interdependent structures that cooperatively interact throughout the process [29]. The *Drosophila *contractile ring is comprised of actin, non-muscle myosin II, the regulatory light chain of myosin II, and anillin. Intriguingly, we observed that the Merlin expression pattern was closely associated with the microtubules of the spindle during cytokinesis. Given the fact that Merlin interacts with microtubule, actin, and myosin heavy chain, it is possible that Merlin may be involved in the assembly of the spindle and membrane addition. Further colocalization analysis will be needed to better understand the role of Merlin in this meiotic process.

Studies in mammalian cells indicate that Merlin functions both as a growth and tumor suppressor [15]. In *Drosophila*, tumor suppressors often regulate cell proliferation in a tissue-specific manner [30]. Two genes previously reported to have tumor suppressor property in the male gonad are *bam *and *bcgn*. Mutations in these genes result in a large number of cells resembling those in early germline stages [30]. Mosaic analysis in the eye tissue has revealed that *Merlin *mutant clones over-proliferate relative to normal sister clones; however, no tumors were found in the homozygous *Merlin *mutant tissue [8]. This result indicates that *Merlin *mutations belong to the class of overgrowth mutations. Upon careful examination of the *Mer*^3 ^and *Mer*^4 ^testis tissues, we also did not find any tumors.

Cyst polarization involves nuclear migration to a defined area of the cyst wall [3]. Although specific details about cyst polarization are not understood, it is envisaged that nuclear migration requires the participation of the actin cytoskeleton. Since Merlin interacts and modulates the actin cytoskeleton and other cytoskeletal apparatus, *Merlin *mutations could affect nuclear migration and lead to spermatid nuclei in two disorganized groups or more scattered in the mutant cysts as we have observed. Alternatively, the presence of Merlin in the Nebenkern suggests that Merlin may play a role in cyst polarization through Nebenkern. Nevertheless, *Merlin *represents the first gene whose mutation affects cyst polarization in spermatogenesis. Previously, in a genetic screen for genes functioning in embryonic axis specification, MacDougall et al. [26] found that *gurken *(*grk*) mRNA localization is altered in Mer mutant embryos. Normally, Grk signal instructs about 200 follicle cells to adopt a posterior fate. In turn, the posterior follicle cells send a polarizing signal back to the oocyte. Consequently, it induces the reorganization of oocyte microtubules, determining the localization of different mRNA and oocyte nuclear migration in the oocyte. Despite a broad expression pattern of Merlin in the egg chamber [17], *Merlin *appears to be specifically required non-autonomously only in a small group of follicular cells to maintain the polarity of posterior follicle cells and to limit their proliferation [26]. This finding is consistent with our data showing Merlin also has a role in cyst polarization during the spermatid pre-elongation period.

It should be mentioned that *Merlin *mutations also affect nuclear shaping during spermatid elongation as we observed a few spermatids with a round but not needle-shaped head in the mutant cysts. Recent studies [31-33] have suggested a possible contribution of acrosome to nuclear shaping because defective acrosome development leads to round-headed sperm in mice. The biogenesis of the acrosome, a derivative of the Golgi complex important for sperm-egg penetration, requires the formation of the transient microtubule-containing manchette caudally to the acrosome [34]. In addition, the assembly of an F-actin-containing cytoskeletal plate, called acroplaxome, serves as an anchor for the developing acrosome to the nuclear envelope [35]. Given the well-established relationship between Merlin and the actin filament or microtubule, *Merlin *mutations may affect any of the cytoskeleton-mediated structures required for acrosome formation. It is interesting to note that we have detected intense Merlin staining in the acrosomal region, suggesting a possible role of Merlin in this organelle.

Actin is a major cytoskeletal component of the IC, and individualization is accomplished by the assembly of the cytoskeletal-membrane complex at the nuclear end of the cyst [1,4]. Our results showing scattered nuclei and dispersed actin cones found in the *Merlin *mutant cysts are consistent with the idea that spermatid nuclei provide a physical scaffolding for the assembly of the IC. Intriguingly, several *Drosophila *mutants with scattered nuclei, including *Chc*^4 ^(*Clathrin heavy chain*), *scat*^1 ^(*scattered*), *cbx*^05704 ^(*crossbronx*), *EcR*^06410 ^(*Ecdysone Receptor*), also display the dispersed IC phenotype [4]. The gene *Clathrin heavy chain *has been shown to participate in a number of biological processes, including receptor-mediated endocytosis, neurotransmitter secretion, and sperm individualization. The gene *scattered *is involved in Golgi-to-vacuole transport, retrograde transport from endosome to Golgi, and spermatid individualization. The gene *crossbronx*, encoding a ubiquitin-protein ligase, is important for the ubiquitin cycle and spermatid individualization. The gene *Ecdysone Receptor*, whose protein product responds to hormone stimuli, is essential for embryonic development and organogenesis, including spermatid development. Curiously, Merlin has been shown to promote endocytosis of several signaling receptors [24,25]. However, how this set of genes is connected to the individualization process remains to be determined.

Spermatid individualization involves membrane remodeling and the outcome of this process is endowing each spermatid with its own plasma membrane and simultaneously removing most of the syncytial cytoplasm from between sperm tails as it proceeds [33,36]. At present, we do not know how *Merlin *mutations lead to excessive cytoplasmic remnants and poorly organized spermatids at the end of individualization. Although Merlin may have a role in membrane remodeling as previously suggested, it is possible that spermatogenesis is such an orchestrated process that perturbation in each stage results in specific abnormalities, which could subsequently affect the following events.

Merlin has been shown to localize underneath the plasma membrane at cell-cell junctions and other actin-rich sites [10,11]. The detection of a high concentration of Merlin protein in the Nebenkern at the onion stage and its maintenance throughout mature sperm formation imply a unique role of Merlin in mitochondria formation and function. The presence of two or multiple paracrystalline bodies in the major mitochondrial derivative of *Merlin *mutant spermatids could result from Merlin dysfunction, leading to such an abnormal Nebenkern structure. A similar abnormality has also been observed in the mutants defective in the gene *fuzzy onion *(*fzo*), encoding a GTPase [37] or *rotund *(*rn*), coding for a Rac GTPase activating protein [38]). However, it is not known whether Merlin function links to these signaling molecules in regulating Nebenkern formation. One fundamental function that mitochondria provide is the production of ATP, which serves as an energy source. The Nebenkern structure is pivotal to sperm motility. Although rare sperm could still be found in the *Mer*^3 ^testis, it is likely that without normal Merlin function, sperm motility is impaired.

It should be mentioned that Merlin is not the only growth suppressor whose loss results in male sterility. Like Merlin, the Tumor Suppressor for Lung Cancer 1 (TSLC1) protein, an immunoglobulin superfamily molecule predominantly expressed in the brain, lung and testis, plays important roles in cell adhesion and tumor invasion in mammals [39]. Interestingly, TSLC1-deficient mice also produce round spermatids and are sterile. Thus, it will be important to see whether Merlin deficiency in the testis has any effect on the fertility of mice.

## Conclusion

*Drosophila Merlin *mutant flies are viable but sterile, and the sterility phenotype is rescued by the introduction of a wild-type *Merlin *gene. *Merlin *mutations affect meiotic cytokinesis of spermatocytes, cyst polarization and nuclear shaping during spermatid elongation, and spermatid individualization. The Merlin protein is enriched in the Nebenkern and this mitochondrial localization is maintained throughout sperm formation. These results suggest a role of Merlin in mitochondria formation and function during various stages of spermatogenesis. Further investigation of the action of Merlin in mitochondria is warranted.

## Methods

### Fly stocks

Flies were maintained at 25°C in standard cornmeal yeast-agar medium. Various *Merlin *mutant strains were generously provided by Rick G. Fehon at University of Chicago, Chicago, IL [40]. Hemizygous *Mer*^3 ^males were taken from the strain *w Mer*^3^*P{ry [+t7.2] = neoFRT}19A/FM6, y B*. The *FM6, y B *siblings and the *y w *males from the stock *y w Pim 19A-FRT/TM6, Tb *(abbreviated here as Pim) were used as controls. The strain *y w Mer*^4^*P{ry [+t7.2] = neoFRT}19A/FM7i, P{w [+mC] = ActGFP}JMR3 *was used as a source of *Mer*^4 ^mutant individuals. The *w sn*^3^***l**(1)18D**Eb**^**3**^ P{ry [+t7.2] = neoFRT}19A; P{w [+mC] = cosMer*^+^*}3/+ *strain with an insertion of a genomic fragment containing the entire *Mer *gene was also used. Transgenic strains carrying the pUAST-*MycMer*^+ ^or pUAST-*MycMer*^3 ^constructs have been described previously [8]. The strain, containing an insertion of a transposable element carrying the green fluorescent protein (GFP) tag inserted into the *CG8351 *gene, was kindly provided by Alain Debec of Université Pierre et Marie Curie, Observatoire Océanologique, Villefranche-sur-mer, France. This strain allowed labeling the cytoplasm of all cells uniformly during spermatogenesis. The strain *y w; Ki Delta2-3 *carrying endogenous transposase activity was a gift from the laboratory of Igor Zhimulev, Institute of Cytology and Genetics, Novosibirsk, Russia.

### Acetic acid/orcein and DAPI staining

The females *w Mer*^3^*P{ry [+t7.2] = neoFRT}19A/FM6, y B *were mated with the males *FM6, y B*/*Y *from the same stock. The resulting *Mer*^3 ^male *y w Mer*^3^*P{ry [+t7.2] = neoFRT}19A/Y *and *control FM6, y B/Y *males were obtained and their testes were dissected. The squashed preparations of testes were performed according to Ashburner [27]. Briefly, testes were dissected in Hanks balanced salt solution (HBSS) and stained in a 1:1 mixture of 1% acetic acid/orcein and 1% acetic acid/carmine for 2 hours at room temperature. Stained testes were examined under phase-contrast optics of an Axiovert-200 microscope (Carl-Zeiss). For DAPI staining, dissected testis tissues were fixed in 3.7% formaldehyde in Dulbecco's phosphate-buffered saline (PBS), pH 7.2, and stained with DAPI (1.5 μg/ml) prior to visualization under the epifluorescence optics of an Axiovert-200 microscope.

### Transgenesis

Genomic DNA were isolated from flies carrying the *UAS-MycMer*^+ ^or *UAS-MycMer*^3 ^insertion as described above and amplified by PCR to generate *Merlin *cDNAs as previously described [8]. The *Merlin *cDNAs were inserted into the pUASP vector at the *Sac*II and *Xba*I sites. The DNA inserts in the plasmids were confirmed by restriction digestions and direct sequencing. Embryos with the genetic constitution *y w; Ki Delta2-3*, carrying the endogenous transposase *Delta2-3*, were injected with the pUASP-*MycMer*^+ ^or pUASP-*MycMer*^3 ^DNA. After reaching the adult stage, the injected flies were mated with *y w *mating partners. The resulting *w*^+ ^progeny were isolated and crossed to establish a strain carrying a transposition. A few independent insertions were obtained for each construct, and the presence of the Merlin coding sequence in the transposants was tested by PCR analysis of genomic DNA described above.

### Living cytology

For the examination of sperm motility, seminal vesicles of male flies that had been alone for three days were isolated and checked for movement of sperm heads under Varel contrast optics of an Axiovert-200 microscope. For general spermatogenesis inspection, dissected testes were squashed in HBSS using coverslips as described by Fuller [2]. The unfixed preparations of live cysts were examined for the coiling process according to Cross and Shellenbarger [3].

### Antibody staining

Dissected testes were placed onto poly-L-lysine coated slides. To isolate cysts, the dissected testes were pierced using a tungsten needle attached to a Narishigi micromanipulator. Slides with testis tissues attached were fixed in 3.7% formaldehyde in PBS, pH 7.2. After washing in PBS 10 min three times, fixed tissues were permeated with 1% Triton X-100 in PBS for 30 min and then pretreated with the blocking solution containing 1% non-fat dry milk in PBS. Pretreated tissues were incubated with a guinea pig anti-Merlin antibody (1:6000 dilution; [17]) or a mouse anti-α-tubulin antibody (1:500 dilution; Sigma Chemicals) overnight. After washing with PBS three times, a secondary antibody conjugate (Alexa 488-conjugated anti-guinea pig IgG [1:700 dilution], Alexa 568-conjugated anti-mouse IgG [1:200 dilution, or a FITC-conjugated anti-mouse IgG [1:50 dilution]) was added for 2 hours at room temperature. To visualize actin filaments, FITC-conjugated phalloidin (1:50 dilution; Molecular Probe) was used. In some experiments, nuclei were stained with DAPI (1.5 μg/ml). After staining, the slides were mounted in Mowiol with 10% DABCO and examined under the epifluorescence or phase-contrast optics of an Axiovert-200 or an Olympus BX50 microscope.

### Electron microscopy

Dissected testes were fixed in 2% glutaraldehyde in PBS, pH 7.4, for 2 hours and then treated with 1% osmium tetraoxide in PBS for 1 hour. Treated tissues were stained with 1% uranyl acetate at 4°C overnight. Following dehydration in ascending ethanol solutions, stained tissues were embedded in Agar-100, mounted in the block, and polymerized at 60°C for two to three days. Ultrathin sections were prepared and contrasted by incubating in 1% uranyl acetate and lead citrate, and examined using a Hitachi H7650 or a JEOL 1000SX transmission electron microscope.

## Abbreviations

IC, individualization complex

ERM, the ezrin, radixin, and moesin proteins

*NF2*, the *Neurofibromatosis 2 *gene

FERM, protein 4.1, ezrin, radixin, and moesin

*D-Mer*, the *Drosophila Merlin *gene

*grk*, the *gurken *gene

Grk, the Gurken protein

*Chc*, the *Clathrin heavy chain *gene

*scat*, the *scattered *gene

*cbx*, the *crossbronx *gene

*EcR*, the *Ecdysone Receptor *gene

*fzo*, the *fuzzy onion *gene

*rn*, the *rotund *gene

TSLC1, the Tumor Suppressor for Lung Cancer 1 protein

GFP, green fluorescent protein

HBSS, Hanks balanced salt solution

PBS, phosphate-buffered saline

## Authors' contributions

NVD performed immunostaining analysis, EMA and NVG carried out electron microscopy, SAK conducted genetic crosses, OSY generated pUASP constructs, LVO helped with cytological analysis and prepared a draft of the manuscript, and LSC was the principal investigator of the project and participated in the design, coordination, and writing of the manuscript. All authors read and approved the final manuscript.
